# Survival and Hospital Discharge Achieved Through Intensive Circulatory, Fluid, and Respiratory Support in Severe TAFRO (Thrombocytopenia, Anasarca, Fever, Reticulin Fibrosis, and Organomegaly) Syndrome With Multiorgan Dysfunction: A Case Report

**DOI:** 10.7759/cureus.81952

**Published:** 2025-04-09

**Authors:** Yu Masui, Masafumi Idei, Nobuyuki Yokoyama, Masashi Yokose, Shunsuke Takaki

**Affiliations:** 1 Department of Anesthesiology and Intensive Care Medicine, Yokohama City University Hospital, Yokohama, JPN

**Keywords:** case report, hemodialysis, intensive care, renal dysfunction, septic shock, steroid therapy, tafro syndrome

## Abstract

Thrombocytopenia, anasarca, fever, reticulin fibrosis, and organomegaly (TAFRO) syndrome is an extremely rare and potentially life-threatening inflammatory disorder of unknown cause and sometimes necessitates intensive care due to complex pathological conditions, including respiratory failure, renal dysfunction, circulatory failure, and infection. However, reports detailing intensive care management of TAFRO syndrome remain limited. A 65-year-old woman was diagnosed with TAFRO syndrome, presenting with progressive edema, significant pleural and ascitic effusions, thrombocytopenia, fever around 37.5-38°C, acute kidney injury, and Castleman disease-like features on lymph node biopsy. She was admitted to the intensive care unit (ICU) for management. Treatment included pulse steroid therapy, careful fluid management with frequent assessment of intravascular volume, and hemodialysis. As a result of pleural effusion drainage and high-flow nasal cannula therapy for respiratory failure due to massive pleural effusion, oxygenation and dyspnea improved, allowing for the avoidance of tracheal intubation. Antibiotics and vasopressors were administered to address septic shock caused by a catheter-associated urinary tract infection (CAUTI). With intensive circulatory management and antimicrobial therapy, the patient’s renal function and septic shock gradually improved. Following 37 days of ICU management, the patient was discharged to the general ward. This case highlights the successful intensive care management of a patient with severe TAFRO syndrome and multiorgan dysfunction, achieved through intensive circulatory, fluid, and respiratory management in the ICU despite the complexity of the condition.

## Introduction

Thrombocytopenia, anasarca, fever, reticulin fibrosis, and organomegaly (TAFRO) syndrome, first described in 2010 [1], is an extremely rare acute systemic inflammatory disease characterized by immune dysregulation and cytokine storm. In TAFRO syndrome, cytokine abnormalities are associated with thrombocytopenia; anasarca, defined as severe, generalized edema involving subcutaneous tissue and body cavities such as pleural effusion and ascites; fever and systemic inflammatory reactions; reticulin fibrosis, a pathological finding characterized by increased deposition of reticulin fibers in the bone marrow; and organomegaly, including hepatomegaly and splenomegaly [1-8]. These diverse manifestations are believed to result from excessive production and activity of inflammatory mediators driven by abnormal interleukin-6 (IL-6) signaling [4,5]. IL-6-mediated activation of the Janus kinase (JAK)-signal transducer and activator of transcription (STAT) pathway promotes further production of inflammatory cytokines and aberrant lymphocyte activation, ultimately leading to systemic organ dysfunction. Additionally, IL-6-induced expression of vascular endothelial growth factor (VEGF) increases vascular permeability, while bone marrow fibrosis contributes to impaired platelet production. Nevertheless, many aspects of the disease pathophysiology remain poorly understood [1-8].

Patients with TAFRO syndrome sometimes suffer from multiorgan failure due to renal failure, respiratory failure, cardiac failure, infection, and hemorrhagic complications, and the mortality rate of TAFRO syndrome is approximately 30% [1,2,5]. The mainstay of treatment is immunosuppressive therapy to inhibit excessive IL-6 production and cytokine storms, typically including corticosteroids and tocilizumab; however, approximately 30-50% of cases exhibit resistance to initial treatment [3-7]. Therefore, comprehensive systemic management addressing complications such as renal failure, respiratory failure, and infections is crucial alongside control of the underlying disease [9,10].

Herein, we report a case of TAFRO syndrome managed in the intensive care unit (ICU). The patient received immunosuppressive therapy, including pulse steroid therapy, along with fluid management for severe systemic edema, renal replacement therapy for acute kidney injury, respiratory support, avoiding tracheal intubation through pleural drainage and high-flow nasal cannula (HFNC) therapy for respiratory failure due to massive pleural effusion, and circulatory support with vasopressors for septic shock. The patient showed improvement in inflammatory response and edema, avoided mechanical ventilation, recovered from acute kidney injury, and was ultimately discharged alive from the hospital.

## Case presentation

A 65-year-old woman (150 cm, 73.5 kg) presented to her primary care physician with complaints of abdominal pain, persistent low-grade fever around 37.5-38.0°C, and progressive edema of the extremities for approximately two weeks. The patient’s medical history included type 2 diabetes mellitus managed with dietary therapy and colorectal polyps. There was no notable family history. As a gastrointestinal infection was suspected by the referring physician, ceftriaxone was administered intravenously at a dose of 2 g per day. However, the patient’s abdominal pain and fever persisted, and the edema did not improve. She was therefore referred to our hospital and admitted for further evaluation and treatment.

**Figure 1:**
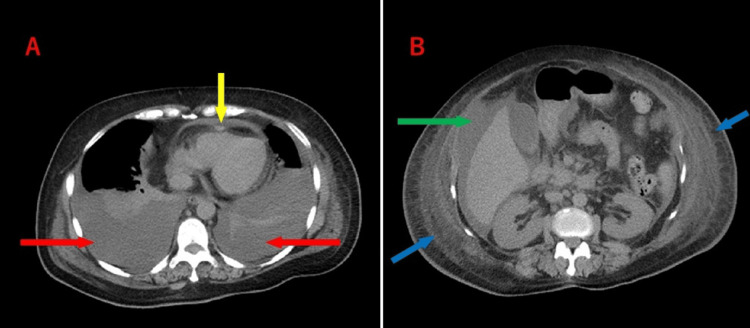
Computed tomography (CT) scan captured at admission to our hospital (A) Chest CT image showing massive bilateral pleural effusion (red arrows) and pericardial effusion (yellow arrow). (B) Abdominal CT image showing ascites (green arrow) and significant edema of subcutaneous tissue (blue arrows).

A computed tomography (CT) scan performed after admission revealed massive bilateral pleural effusion, pericardial effusion, ascites, subcutaneous tissue edema, axillary, mediastinal, and periaortic lymphadenopathy, and bilateral adrenal enlargement (Figure 1).

On admission, the patient's vital signs were as follows: alert and oriented, blood pressure 112/64 mmHg, heart rate 78 beats per minute, respiratory rate 18 breaths per minute, oxygen saturation 94% on room air, and body temperature 37.6°C. Her physical examination revealed weight gain (10 kg in one month), abdominal distention (soft, no tenderness), and marked indurated edema of the extremities. Laboratory findings showed marked hypoalbuminemia, elevated levels of serum creatinine, C-reactive protein (CRP), alkaline phosphatase (ALP), and soluble interleukin-2 receptor (sIL-2R). Antinuclear antibodies were not elevated (Table 1).

**Table 1:** Laboratory data on admission. Abnormal values are indicated in bold. WBC, white blood cell; RBC, red blood cell; CK, creatine kinase; AST, aspartate aminotransferase; ALT, alanine aminotransferase; LDH, lactate dehydrogenase; ALP, alkaline phosphatase; BUN, blood urea nitrogen; CRP, C-reactive protein; TSH, thyroid stimulating hormone; fT3, free triiodothyronine, fT4; free thyroxine, PT-INR; prothrombin time-international normalized ratio; APTT, activated partial thromboplastin time, FDP, fibrinogen degradation products; ESR, erythrocyte sedimentation rate; sIL-2R, soluble interleukin-2 receptor; IL-6, interleukin-6

Laboratory parameter	Value	Reference range	Unit
WBC	13.7	3.3-8.6	×10^3^/μL
RBC	4.76	3.86-4.92	×10^6^/μL
Hematocrit	41	35.1-44.4	%
Platelet	38.2	158-348	×10^3^/μL
Total protein	5.3	6.6-8.1	g/dL
Albumin	1.6	4.1-5.1	g/dL
CK	91	41-153	U/L
AST	28	13-30	U/L
ALT	20	38-113	U/L
LDH	189	124-222	U/L
ALP	588	38-113	U/L
Total bilirubin	0.5	0.4-1.5	mg/dL
BUN	18	8-20	mg/dL
Creatinine	1.27	0.46-0.79	mg/dL
Na	139	138-145	mmol/L
K	3.8	3.6-4.8	mmol/L
Cl	105	101-108	mmol/L
Ferritin	229	10-120	ng/mL
CRP	26.83	<0.3	mg/dL
TSH	4.78	0.5-5.0	μIU/mL
fT3	1.39	2.3-4.0	pg/mL
fT4	0.82	0.9-1.7	ng/dL
PT-INR	1.26	0.87-1.15	INR
APTT	34.1	25.0-35.0	sec
Fibrinogen	676	186-385	mg/dL
FDP-Dimer	10.12	<0.7	μg/mL
ESR	82	3-15	mm/hr
sIL-2R	1287	145-519	U/mL
IL-6	48.9	<7.0	pg/mL

Serological and antigen tests for Epstein-Barr virus (EBV), human immunodeficiency virus (HIV), human herpesvirus 8 (HHV-8), and severe acute respiratory syndrome coronavirus 2 (SARS-CoV-2) were all negative.

On hospital day 5, biopsies of the left axillary lymph node and bone marrow were performed. Pathological examination of the lymph node specimen revealed findings consistent with Castleman disease, including atrophic germinal centers, thickened mantle zones of lymphoid follicles, and increased interfollicular vascularity. Bone marrow biopsy showed reticulin fibrosis and megakaryocytic hyperplasia. The platelet count gradually declined to 6.6 × 10³/μL, CRP elevated to 21.41 mg/dL, and creatinine increased to 1.54 mg/dL. In conjunction with other clinical findings and established diagnostic criteria (discussed later) [3], the patient was diagnosed with TAFRO syndrome.

Based on elevated IL-6 levels and rapid clinical deterioration, tocilizumab at a dose of 8 mg/kg was initiated on hospital day 13. However, despite anti-IL-6 therapy, no improvement was observed; the platelet count remained at 7.1 × 10³/μL, CRP was 22.54 mg/dL, and creatinine was 1.60 mg/dL. Therefore, methylprednisolone at 160 mg/day was initiated on hospital day 16.

**Figure 2:**
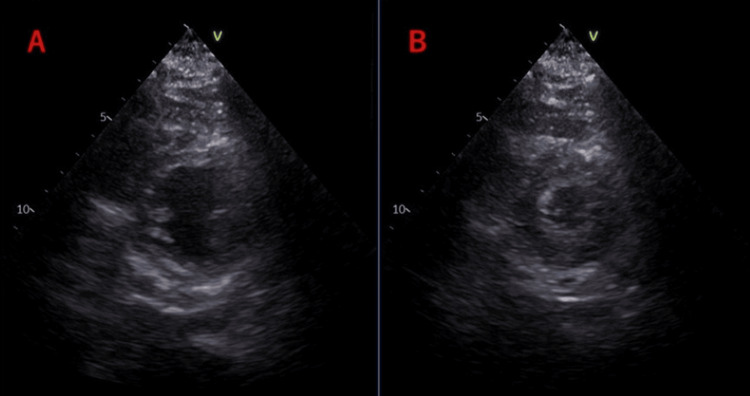
Echocardiography on day 18 of hospitalization (A: diastole, B: systole) Left ventricular diastolic and systolic diameters were 39.4 mm and 19.6 mm, respectively, and ejection fraction (EF) was 82.1%. Wall motion was hyperdynamic, suggesting intravascular hypovolemia.

On hospital day 18, the patient's dyspnea and edema worsened, and echocardiography revealed a narrowing of the left ventricular diameter (Figure 2).

Based on these findings, worsening fluid retention and intravascular hypovolemia were diagnosed, and the patient was admitted to the ICU on the same day. While receiving oxygen at 5 L per minute via face mask, the patient had respiratory rate of 22 breaths per minute, peripheral oxygen saturation (SpO₂) of 93%, and partial pressure of oxygen (PaO₂) of 69 mmHg. Laboratory tests showed a platelet count of 7.3 × 10³/μL, a CRP level of 22.62 mg/dL, and a creatinine level of 1.67 mg/dL. The Acute Physiology and Chronic Health Evaluation II (APACHE II) and Sequential Organ Failure Assessment (SOFA) scores were 15 and 5, respectively.

Pulse steroid therapy with methylprednisolone at 1 g/day was initiated on the same day. In parallel, bilateral pleural drainage of approximately 1000 mL per side and HFNC therapy (flow rate: 50 L per minute, oxygen concentration: 50%) were administered to treat respiratory failure due to massive pleural effusion. These interventions led to an improvement in PaO₂ from 69 to 102 mmHg.

Circulatory and fluid management in TAFRO syndrome is particularly challenging due to fluid shifts into the third space caused by increased vascular permeability. Therefore, during treatment, intravascular volume status and fluid responsiveness were frequently assessed using echocardiography, dynamic indices such as passive leg raising (PLR), and central venous pressure (CVP) measurements. At ICU admission, despite generalized anasarca, echocardiography revealed a collapsed inferior vena cava (IVC), indicating intravascular volume depletion secondary to increased vascular permeability. This necessitated the administration of albumin and blood products. Specifically, blood transfusions were actively administered to correct hypoalbuminemia and anemia when cardiac output increased by more than 10-15% during the PLR. To prevent the worsening of edema due to intravascular volume overload, diuretics were administered when CVP exceeded approximately 10 mmHg. Echocardiography was performed several times daily to measure the diameters of the IVC and left ventricle and to assess cardiac systolic and diastolic function. After completing a three-day course of pulse steroid therapy, methylprednisolone was reinitiated at a reduced dose of 160 mg/day, starting on hospital day 21.

After admission to the ICU, the patient's creatinine and blood urea nitrogen (BUN) worsened to 2.0 mg/dL and 109 mg/dL, respectively, and urine output decreased to 500 mL/day despite diuretic therapy. Therefore, intermittent hemodialysis was initiated on hospital day 28. Following pulse steroid therapy, maintenance immunosuppressive treatment, and hemodialysis, the patient’s condition improved by hospital day 30, with a platelet count of 10.9 × 10³/μL, a CRP level of 10.77 mg/dL, and creatinine decreased to 1.36 mg/dL.

On hospital day 35, the patient developed septic shock secondary to a catheter-associated urinary tract infection (CAUTI) caused by *Klebsiella pneumoniae*. Her systolic blood pressure dropped to 60 mmHg, and the APACHE II and SOFA scores increased to 20 and 9, respectively, indicating clinical deterioration. Empirical antimicrobial therapy with meropenem (1 g every 12 hours) was initiated and continued. Balanced crystalloids, continuous intravenous noradrenaline (up to 0.2 µg/kg/minute), and vasopressin (up to 0.02 units/minute) were administered to maintain hemodynamic stability. Lactate peaked at 2.1 mmol/L (reference range: <1.8 mmol/L) on hospital day 35 and subsequently decreased to 0.9 mmol/L following fluid resuscitation and antibiotic therapy by the next day.

By hospital day 38, the patient’s CRP level had decreased to 2.41 mg/dL, serum creatinine had improved to 0.54 mg/dL, and urine output had increased to 1800 mL/day, allowing for the discontinuation of hemodialysis. The patient recovered from septic shock, with vasopressin discontinued on hospital day 45 and noradrenaline discontinued on hospital day 48. The generalized edema gradually improved, and oxygen therapy was no longer required. By hospital day 54, the platelet count had increased to 9.0 × 10³/μL, CRP had decreased to 0.83 mg/dL, and creatinine had decreased to 0.61 mg/dL. The APACHE II and SOFA scores at that time were 16 and 5, respectively, and the patient was transferred from the ICU to the general ward.

On hospital day 66, the patient developed varicella-zoster virus meningitis, which required treatment with acyclovir and subsequent rehabilitation. She was eventually discharged on hospital day 132 with a platelet count of 22.4 × 10³/μL, CRP of 0.02 mg/dL, creatinine of 0.48 mg/dL, no residual anasarca, and the ability to ambulate independently.

## Discussion

This was a case of severe TAFRO syndrome requiring intensive care management for generalized edema, acute kidney injury, respiratory failure, and septic shock.

In 2010, Tanaka et al. first described three cases of an acute systemic inflammatory disease of unknown etiology, which were later diagnosed as TAFRO syndrome, characterized by hepatosplenomegaly and lymphadenopathy [1]. An epidemiological study in Japan estimated the annual incidence of TAFRO syndrome to be 0.9-4.9 cases per million population [8]. In the cohort analyzed by Nishimura et al., most patients were of Asian descent, particularly Japanese, although cases have also been reported among Caucasian and Hispanic individuals, indicating increasing global recognition of the disease [11]. The median age at onset ranged from the late 40s to mid-60s, with no apparent sex predilection. Accordingly, the patient described in this report falls within the typical demographic profile for TAFRO syndrome. To date, no definitive genetic predisposition has been identified, and no specific underlying conditions or prior medical history have been clearly associated with the development of the disease [11]. TAFRO syndrome is considered a clinical variant related to Castleman disease, which is also thought to result from dysregulated IL-6 signaling. However, due to a more pronounced cytokine storm, TAFRO syndrome tends to present with more severe clinical features, including rapid progression, generalized fluid retention, severe renal dysfunction, and marked thrombocytopenia [3-7].

According to the validated international diagnostic criteria proposed by Nishimura et al., a definitive diagnosis of TAFRO syndrome requires the presence of all four core clinical features: thrombocytopenia (pre-treatment platelet count ≤100 × 10³/μL), anasarca (pleural effusion, ascites, or subcutaneous edema), fever or hyperinflammatory status (body temperature ≥37.5°C of unknown origin or CRP ≥2.0 mg/dL), and organomegaly (small-volume lymphadenopathy in two or more regions, hepatomegaly, or splenomegaly) [11]. In addition, lymph node histopathology consistent with idiopathic multicentric Castleman disease, such as atrophic germinal centers, concentric mantle zones, and interfollicular hypervascularity, is required. At least one of the following supportive findings must also be present: renal dysfunction (estimated glomerular filtration rate (eGFR) ≤60 mL/minute/1.73 m², serum creatinine >1.1 mg/dL in female patients or >1.3 mg/dL in male patients, or the requirement for dialysis), or characteristic bone marrow findings such as reticulin fibrosis or megakaryocytic hyperplasia. Furthermore, exclusion criteria must be applied to rule out infectious, autoimmune, and malignant diseases that can present with similar clinical features [11].

The severity of TAFRO syndrome is assessed based on the extent of anasarca, thrombocytopenia, fever or systemic inflammation, and renal dysfunction [3]. According to these criteria, our patient was classified as having severe TAFRO syndrome.

Patients with TAFRO syndrome may require intensive care for various complications, including respiratory failure secondary to generalized edema and large-volume pleural and peritoneal effusions, renal dysfunction, infections related to immunosuppressive therapy, and septic shock. However, due to the rarity of the disease, few cases have been reported in which patients with TAFRO syndrome received intensive care management [9,10]. José et al. reported a case of a 61-year-old woman with TAFRO syndrome who was successfully treated with intensive immunosuppressive therapy, including methylprednisolone, tocilizumab, and rituximab, along with ICU support involving mechanical ventilation, vasopressor administration, and continuous renal replacement therapy (CRRT) [9]. Similarly, Matsuhisa et al. described a case of a 58-year-old man with TAFRO syndrome who, after receiving intensive immunosuppressive therapy, developed treatment-related complications, including methicillin-resistant *Staphylococcus aureus* bacteremia, *Stenotrophomonas maltophilia* peritonitis, and disseminated candidiasis, ultimately resulting in death from septic shock.

These reports, together with our case, emphasize that in patients with severe TAFRO syndrome, comprehensive intensive care management is as important as immunosuppressive therapy, such as pulse corticosteroids and tocilizumab, for achieving favorable outcomes. This includes circulatory and fluid management, respiratory support, and sepsis management, all of which likely contributed to the patient’s survival. A summary of organ involvement in TAFRO syndrome and the corresponding management strategies is provided in Table 2.

**Table 2:** Intensive care management for organ-specific problems in thrombocytopenia, anasarca, fever, reticulin fibrosis, and organomegaly (TAFRO) syndrome HFNC, high-flow nasal cannula therapy; NPPV, non-invasive positive pressure ventilation

System	Problem	Management in intensive care
Respiratory system	increased extra-vascular lung water	fluid management
	pleural effusion	drainage
	respiratory insufficiency	HFNC/NPPV/mechanical ventilation as needed
Circulatory system	hypovolemia/edema	fluid administration
	septic shock due to infection	treatment of septic shock
Renal system	renal dysfunction (renal/prerenal)	renal replacement therapy as needed
Hepatic system	hepatosplenomegaly	monitoring of liver function
Hematological system	anemia	transfusion
	thrombocytopenia	transfusion
Metabolism and endocrine system	hyperglycemia due to steroid administration	insulin therapy
Infection	immunosuppression with steroid and immunosuppressive drugs	prevention and treatment of infectious diseases
Gastrointestinal system	ascites	abdominal drainage as needed
Nutrition	undernutrition	appropriate nutritional therapy
	hypoalbuminemia	albumin transfusion

Patients with TAFRO syndrome often present with fluid retention due to severe systemic inflammation and increased vascular permeability [1-8]. Although fluid resuscitation is necessary to correct intravascular dehydration and blood transfusions are indicated for anemia, excessive administration of fluids and blood products may result in intravascular volume overload, leading to worsening edema, pleural effusion, and ascites.

In the present case, to prevent circulatory failure due to hypovolemia and avoid exacerbation of edema or anasarca caused by volume overload, the patient was managed in the ICU with frequent assessment of intravascular volume status and fluid responsiveness. These assessments were conducted using echocardiography, dynamic indices including PLR, and CVP monitoring. Given the narrow therapeutic window for fluid balance in TAFRO syndrome, meticulous volume management in the ICU appears to be critical for optimizing outcomes. To address respiratory failure related to pleural and ascitic effusions, chest drainage and HFNC therapy were initiated in the ICU, allowing mechanical ventilation to be avoided.

Patients with TAFRO syndrome require accurate diagnosis and prompt initiation of treatment due to the potential for rapid systemic deterioration. However, no standardized evidence-based treatment regimen has been established to date [3-6]. Empirically, corticosteroids (1 mg/kg for two weeks), cyclosporine A, anti-IL-6 receptor antibodies, and thrombopoietin receptor agonists are commonly used [3-7]. In addition, some case reports have suggested that plasma exchange and chemotherapeutic regimens such as cyclophosphamide, doxorubicin, vincristine, and prednisolone may be effective in certain cases [3-7]. Nevertheless, even with these treatments, severe cases that are refractory to therapy may progress to multiple organ failure and result in death.

The patient initially presented with mild thrombocytopenia and was preemptively treated with tocilizumab due to concerns regarding steroid-induced immunosuppression. However, because laboratory parameters such as platelet count, inflammatory markers, and renal function showed limited improvement, corticosteroids were subsequently added. Pulse steroid therapy proved effective in controlling the disease in combination with systemic management in the ICU and was considered a key factor contributing to the patient's survival and hospital discharge despite the severity of illness.

While aggressive immunosuppressive therapy is essential for disease control, it may predispose patients to serious complications, including infections and sepsis, which can further exacerbate their condition. In the present case, the patient developed septic shock secondary to CAUTI, likely facilitated by intense immunosuppression. Early recognition of sepsis in the ICU, timely administration of appropriate antimicrobial therapy, and meticulous circulatory management were critical in preventing further organ dysfunction.

## Conclusions

This case involved severe TAFRO syndrome with multiple organ failure; however, in addition to intensive immunosuppressive therapy with pulse steroid therapy and tocilizumab, comprehensive intensive care management including circulatory and fluid management, respiratory support, and treatment of septic shock resulted in the resolution of edema and renal dysfunction, avoidance of mechanical ventilation, recovery from septic shock, and ultimately, survival to hospital discharge without sequelae. To our knowledge, this is a rare reported case in which intensive care was implemented for the management of TAFRO syndrome, and it may provide valuable insights for the future clinical management of similarly severe cases.
